# Influence of the Efavirenz Micronization on Tableting and Dissolution

**DOI:** 10.3390/pharmaceutics4030430

**Published:** 2012-09-12

**Authors:** Eduardo Costa Pinto, Flávia Almada do Carmo, Thiago da Silva Honório, Rita de Cássia da Silva Ascenção Barros, Helena Carla Rangel Castro, Carlos Rangel Rodrigues, Valéria Sant'Anna Dantas Esteves, Helvécio Vinícius Antunes Rocha, Valeria Pereira de Sousa, Lucio Mendes Cabral

**Affiliations:** 1 Department of Pharmaceutics, Faculty of Pharmacy, Universidade Federal do Rio de Janeiro, Av. Carlos Chagas Filho, 373, CCS, Bloco Bss 31, University City, Rio de Janeiro, RJ, CEP: 21.941-902, Brazil; Email: educostapinto@ig.com.br (E.C.P.); honorio_thiago@yahoo.com.br (T.S.H.); rcbarros@pharma.ufrj.br (R.C.S.A.B.); rangel@pharma.ufrj.br (C.R.R.); vsdantas@gmail.com (V.S.D.E.); valeria@pharma.ufrj.br (V.P.S.); lmcabral@pharma.ufrj.br (L.M.C.); 2 Laboratory of Antibiotics, Biochemistry, Institute of Biology, Universidade Federal Fluminense, Campus of Valonguinho, Niterói, RJ, CEP: 24.210-130, Brazil; Email: hcastrorangel@yahoo.com.br; 3 Institute of Drug Technology, Fundação Oswaldo Cruz, Av. Comandante Guaranys, 447, Curicica, Rio de Janeiro, RJ, CEP: 22.775-903, Brazil; Email: helveciorocha@far.fiocruz.br

**Keywords:** AIDS, size distribution, surface area, bioavailability, brick dust drug

## Abstract

The purpose of this study was to propose an analytical procedure that provides the effects of particle size and surface area on dissolution of efavirenz. Five different batches obtained by different micronization processes and with different particle size distribution and surface area were studied. The preformulation studies and dissolution curves were used to confirm the particle size distribution effect on drug solubility. No polymorphic variety or amorphization was observed in the tested batches and the particle size distribution was determined as directly responsible for the improvement of drug dissolution. The influence of the preparation process on the tablets derived from efavirenz was observed in the final dissolution result in which agglomeration, usually seen in non-lipophilic micronized material, was avoided through the use of an appropriate wet granulation method. For these reasons, micronization may represent one viable alternative for the formulation of brick dust drugs.

## 1. Introduction

The development of pharmaceutical solid oral dosage forms for Class II drugs (Biopharmaceutics Classification System) represents one of the biggest challenges facing the pharmaceutical industry [1]. Low water solubility and consequent dissolution are the limiting factors preventing these drugs’ derivative formulations presenting adequate relative bioavailability. This restriction is so severe in some cases that these molecules have been denominated brick dust drugs [2]. As there are often no therapeutic alternatives to these brick dust drugs, not even their conversion into salt or co-crystallization, this problem must therefore be resolved by optimizing the formulation [3]. Efavirenz provides an example of this challenge due its extreme water insolubility. This drug is a non-nucleoside reverse transcriptase inhibitor (NNRTI) used for the treatment of the human immunodeficiency virus (HIV) type 1 infection [4]. Its industrial processing is directly related to its macroscopic properties; adequate dissolution and bioavailability must result from a tablet derived from the compression of 300 or 600 mg of the drug. The micronization of this active pharmaceutical ingredient (API) has been the most viable option to overcome these problems of dissolution [4].

Processes of micronization and co-micronization have successfully been used to optimize the dissolution of poorly soluble drugs, such as glibenclamide [5]. However, the appropriate characterization of physical and mechanical properties obtained after micronization presents itself as the differential between the success and failure of this operation, particularly with drugs of such low water solubility as efavirenz. The determination of the influence of micronization on the surface area, crystal structure, static charge, particle size and particle size distribution and, consequently, on the solubility/dissolution of the micronized material, is important in order to ensure the production of a tablet with adequate bioavailability and processability [6,7,8]. Polymorphism, often regarded as the single most important factor in determining variations in the solubility of a drug, is of little relevance in the case of efavirenz, as the materials available in the market present the same structure. Other forms are cited in literature or even patented but they are not yet available commercially [9,10].

In this work, different batches of efavirenz produced by a Brazilian manufacturer were characterized. The characteristics obtained by two different micronization techniques were compared in order to investigate a possible correlation between the physical-mechanical properties of the micronized material and dissolution values. The aim of this study was the parameterization of the influence of brick dust drug physical properties in the micronization process to predict the optimal tablet formulation containing 600 mg of efavirenz based on the analysis of the API.

## 2. Experimental Section

### 2.1. Materials

The reagents ammonium acetate and sodium lauryl sulfate were acquired from Vetec (Rio de Janeiro, Brazil). The pharmaceutical excipients hydroxypropylcellulose, microcrystalline cellulose (Avicel^®^ PH 101), croscarmelose sodium (Ac-Di-Sol^®^) were purchased from FMC (Brussels, Belgium), spray-dried alpha lactose from Lactose New Zealand (Hawera, New Zealand) and magnesium stearate from Mallinckrodt (Hazelwood, USA). The acetonitrile chromatographic grade was purchased from Tedia (Rio de Janeiro, Brazil). The APIs produced by Nortec Química (Rio de Janeiro, Brazil) and Globe Química (São Paulo, Brazil) were kindling donated by Farmanguinhos Laboratories (Rio de Janeiro, Brazil).The tablets were produced by Farmanguinhos and kindly donated to this study. The efavirenz standard with 100.0% purity was supplied by Globe Química. All solutions were prepared using MilliQ water (Millipore, Bedford, MA, USA).

### 2.2.Micronization

Micronization was carried out at a feed rate of 25 kg/h or 50 kg/h, employing an open system with no further classification, using the Jet Mill J-200 Model, Tecnologia Meccanica (Bergamo, Italy). In each case, the feed rate remained constant throughout the process in order to avoid possible batch-to-batch variations in the particle size distribution of the processed material.

### 2.3. Characterization and Evaluation of Efavirenz Samples

The pH measurement was performed using an Orion pH meter (ION 450 M) (Londrina, Paraná, Brazil). Median diameter (MD) and polydispersity index (PI) measurements were performed by laser diffraction analysis using the Malvern MS2000 Particle Size Analyzer (Worcestershire, United Kingdom). The analysis parameters were: a 90° detection angle, 100 scans over two minutes for each sample, a refractive index of 1.330 and at a temperature of 22 °C using a wet analysis module and a 0.02% aqueous solution of Tween 80 as dispersant. These analyses were carried out in triplicate on three different days. Scanning electron microscope (SEM) analyses were performed with a JEOL 1200EX (Tokyo, Japan)electronic transmission microscope to study structural changes with a vacuum system operating at 10^−4^ torr. The samples were prepared in a volumetric flask with 10 mg of powder. The samples were then sputter-coated with gold/palladium by vacuum evaporation.

The polymorphic content of efavirenz and the calorimetric characteristics of all batches studied were obtained by differential scanning calorimetry (DSC). The samples were analyzed at a nitrogen outflow of 22.5 mL·min^−1^ and heated 0 °C–350 °C (10 °C.min^−1^) using a DSC 60 analyzer (Shimadzu; Tokyo Japan). The samples obtained from different suppliers were also analyzed by X-ray diffractometry (DRX) using the Rigaku Miniflex X-ray Diffractometer (Tokyo, Japan), operated at 30 kV, 15 mA, 0.05 mm, 1°/min, at room temperature, using CuK_2_ radiation (0.15418 nm wavelength). The diffraction range was adjusted according to the main purpose of each analysis. The diffraction of the 2*θ* scan was registered and a diffraction sweep angle of 1° to 50° was used to verify the material crystallinity and polymorphism. The specific surface area (SSA) was measured by gas adsorption using a Micromeritics Gemini VI surface area and pore size analyzer (Micromeritics Instrument Corporation, Georgia, USA). Adsorption measurements were performed with nitrogen as the analytical gas and helium as the reference gas for free space measurements. The Micromeritics SmartPrep degasser was used to degas the samples using nitrogen gas for 24 h at 25 °C prior to analysis. The evacuation was performed at a rate of 500 mmHg/min for 1 min and 10 s (equilibration time for adsorption). The amounts of nitrogen gas adsorbed at a range of relative pressures (0.05 < *p*/*p_0_* < 0.35) were determined in order to calculate the specific surface area by the Brunauer, Emmett and Teller (BET) method.

### 2.4. Drug Characterization

The dissolution was performed by dispersing the raw material using the Vankel VK 750 D dissolution system (Palo Alto, USA). The powder dissolution study is an analytical technique in which the powder sample to be analyzed is inserted into the dissolution vessel after dispersion using 5.0 mL of dissolution media, in this case using USP apparatus II (paddle) and a rotation speed of 50 rpm, as recommended for the dissolution of solid dosage forms [11,12]. Nine hundred milliliter of aqueous sodium lauryl sulfate (SLS) (0.5%, 1.0% and 2.0% *w*/*w*) was used as the dissolution medium, maintained at 37 ± 0.5 °C. Samples of 10 mL were withdrawn at 10 min intervals and replenished with dissolution media for 60 min. The dissolution samples were filtered with a Millipore 0.22 μm PVDF filter prior to UV analysis. The efavirenz calibration curves were used to analyze the samples at 248 nm using six replicates.

The residue of the drug deposited at the bottom of the dissolution vessel was dried and analyzed by XRD and NMR. The study of ^1^H and ^13^C NMR was performed in deuterated DMSO (400 MHz and 50 MHz ^1^H–^13^C) using the Varian MR-400 NMR (Palo Alto, USA).

### 2.5. Tablet Formulation

Efavirenz tablets were prepared by wet granulation. Due to its low solubility, only water was used as the granulation liquid, also following the most updated recommendations for pharmaceutical industry, based on concepts of environmental, operator and structure protection. Table 1 presents the components used for tablet preparation and the quantitative formula is not shown for reasons of commercial confidentiality. The final tablet quality parameters set out in the USP forum were followed [13].

**Table 1:** Formulation of efavirenz batches.

Ingredient	Application
Efavirenz	API
Hydroxypropylcellulose	Binder
Microcrystalline cellulose PH101	Diluent
Croscarmellose sodium	Disintegrant
Spray-dried alpha-lactose	Diluent
Sodium lauryl sulfate	Surfactant
Magnesium stearate	Lubricant

The tablets were prepared with the direct application of water using a peristaltic pump and stainless steel nozzle to spray the water in a pilot high-shear granulator. The wet granulate was sieved in a oscillant granulator and after that it was dried in an oven (at 55 °C until 1.5%–2.0% *w*/*w* moisture). The dried granulate was again normalized in the oscillant granulator (1.0 mm mesh). The dried granulate was mixed with other excipients and submitted to a V mixer pilot 6 L for 10 min, and after that magnesium stearate was also added and the powder was finally blended for a further 5 min. The final mixture was compressed in a Lawes 2000/10 PSC tablet machine (São Paulo, Brazil) with 14-mm round biconcave punches. The amount of water applied in both types of granulation was 5.5 L per 12 kg of powder. In the case of high-shear spray application, the water application rate was around 83.3 mL/min and the end point determined during the granulation process was 6.0 A. Tablet hardness was measured on the Erweka hardness tester (TBH 20, Heusenstamm, Germany), the average of 20 tablets was calculated. Tablet dissolution profiles were carried out following the same procedure as the dissolution test of the efavirenz raw material (powder dissolution).

### 2.6. Drug and Tablet Assay

The efavirenz content in both the raw material and tablet form followed the USP Pharmacopoeia Forum [13]. The analysis was carried out using high performance liquid chromatography (HPLC), with UV-Vis detection at 252 nm and the Hypersil BDS brand (Thermo Scientific, USA) L1 column (4.6 mm × 25 cm).

The mobile phase consisted of a mixture of buffer and acetonitrile (50:50), filtered and degassed. The buffer solution was prepared by dissolving 800 mg of ammonium acetate in 1 L of water, adjusting to pH 7.5 with diluted ammonia solution. The flow rate used was 1.5 mL/min, the injection volume was 20 µL and the retention time was 10.9 min.

A standard solution was prepared from an accurately weighed quantity of the reference chemical substance, using the mobile phase as diluent to obtain a solution of 0.10 mg/mL. The same procedure was followed for the sample preparation; a sample from 20 tablets was crushed, homogenized and weighed to produce the required mass of efavirenz to produce a final concentration of 0.10 mg/mL.

### 2.7. Statistical Data Analysis

One-Way ANOVA and Wilcoxon matched pairs tests were used to analyze all statistical data obtained in this study using Statistica analysis pack (Statsoft; Tulsa, USA). Differences were considered significant if the associated probability level (*p*) was lower than 0.05. Particle size analysis and specific surface area were realized in triplicates and dissolution studies were carried out in six replicates.

## 3. Results and Discussion

Batches EFA 1, 2 and 3 were micronized at a rate of 50 kg/h and batches EFA 4 and 5 at a rate of 25 kg/h. After micronization, particle size distribution was evaluated in all batches and the results are shown in Table 2. A significant difference was observed in the size distribution profiles obtained from different rates of micronization (*p* = 0.00342). 

**Table 2:** Particle size distribution of efavirenz batches 1–5.

Batch	Particle Size Distribution			
	*d*_(0.1)_ μm	*d*_(0.5)_ μm	*d*_(0.9)_ μm	PI
EFA1	2.105	11.656	34.174	2.7513
EFA2	1.352	6.166	28.668	4.4301
EFA3	1.581	7.699	24.967	3.0375
EFA4	1.013	3.443	23.096	6.4139
EFA5	0.975	2.736	7.654	2.4412

*d*_(0.1)_ μm, *d*_(0.5)_ μm and *d*_(0.9)_ μm means particle diameter corresponding to 10%, 50% and 90% of the cumulative distribution, respectively. PI means polydispersity index.

It can be noted in Table 2 that EFA 4 and EFA 5 presented lower values of particle size than the other batches. Reductions in particle size generally mean a higher dissolution in water, which is recommended for brick dust drugs [6,7,14]. The higher polydispersity index observed for EFA 4 indicates a greater variety of particle diameter, leading to potentially lower and erratic dissolution of EFA 4 in relation to EFA 5. When micronization was performed at a rate of 50 kg/h, an increase in the diameter size population (>10 µm) was observed when compared to a rate of 25 kg/h (EFA 4 and 5), at which there were fewer particles in this size range. This result indicates that the rate of 25 kg/h is more suitable for efavirenz micronization. Eventual consequences of the micronization process, such as the loss of bulk flowability and difficulty of compression, become secondary when considered in comparison to the need to increase drug dissolution [15]. These processing problems can be avoided in the formulation process through the wet granulation of the drug before compression [16].

**Figure 1:**
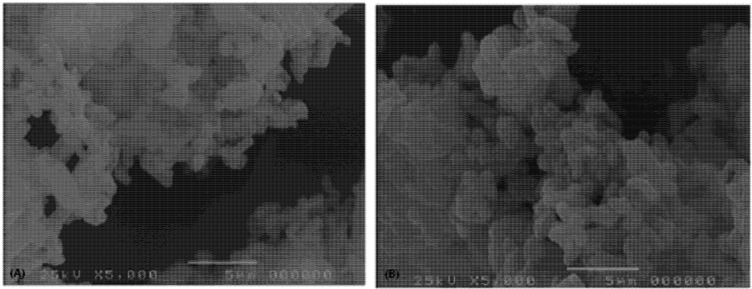
(**A**) Electron scanning microscopy of batches EFA 5; (**B**) EFA 2 obtained from different micronization procedures.

Figure 1 shows the electron scanning microscopy of batches EFA 5 and EFA 2 obtained from different micronization procedures. It can be noted that EFA 5 shows greater uniformity in terms of particle size distribution and geometry (Figure 1 and Table 2).

The scanning electronic microscopy analysis of batch EFA 5 (Figure 1) also shows greater uniformity in terms of particle size distribution when compared to EFA 4, which corroborates the polydispersity index measurement (Table 2), despite the occurrence of agglomeration in both cases. However, the presence of small particles significantly below the average diameter measured by laser diffraction can be observed in both batches. This result indicates that particles may dissolve during laser diffraction analysis leading to results with low reproducibility in terms of average diameter and polydispersity index. The tendency of the material to agglomeration contributes to a greater probability of collapse when compressed or granulated, and which increases in the presence of particles with a diameter of 1 µm or less. This characteristic causes a higher sensitivity to changes in the variables of the manufacturing process in terms of the increase or decrease of tablet dissolution.

**Figure 2:**
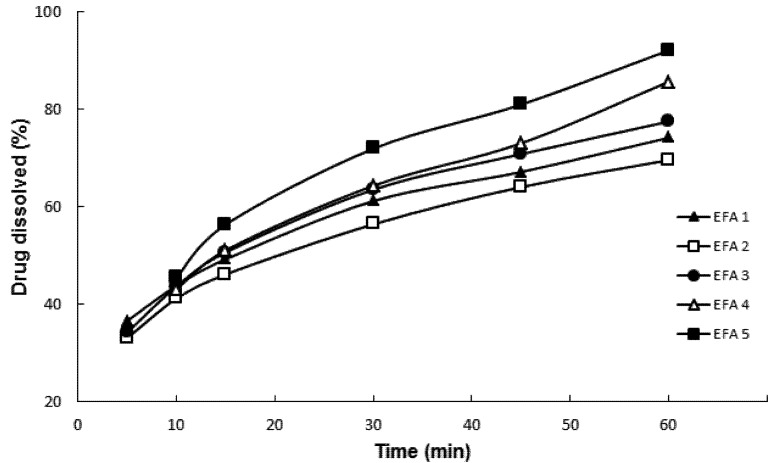
Results of dissolution by dispersion of efavirenz batches 1 to 5 (EFA 1 to 5).

Powder dissolution testing by particle dispersion (Figure 2) was carried out following the parameters of tablet dissolution methodology proposed by the USP Pharmacopeial Forum for efavirenz [13]. Drug dissolution was evaluated in order to predict the impact of physical chemical parameters on dissolution behavior, modulated by its physicochemical properties, of the final formulation. The dissolution media containing water and SLS at 1.0% and 2.0% *w*/*w* was not discriminatory. The dissolution profiles of EFA 1 to 5 were not statistically different (*p* = 0.07623). However, when using 0.5% *w*/*w* SLS, it was possible to obtain statistically different profiles (*p* = 0.00231). This medium was considered ideal for the characterization of the micronized samples indicating the importance of dissolution media choice in brick dust drug formulation.

Figure 2 shows that EFA 4 and EFA 5 micronized at a rate of 25 kg/h presented the highest percentage of drug dissolution after 60 min, with an average and relative standard deviation (RSD) of 85.69% ± 0.44% and 92.14% ± 0.95%, respectively. The batch obtained at 50 kg/h with the highest dissolution at 60 min was EFA 3 with a percentage of 77.52% ± 1.44%. The dissolution test by particle dispersion showed to be suitable for monitoring the solid state of the API to be used in the preparation of its derivative tablets. Different from the punctual analysis (in 60 min), when the dissolution was evaluated by its individual profiles, there were no mathematical differences between the samples according to parameters *f*1 and *f*2. To consider two dissolution profiles as identical, the value of *f*1 should be lower than 15, and the one of *f*2 higher than 50. The profiles were compared one to one and the only difference was assigned when sample EFA2 was compared with EFA5 (*f*1 = 18.45 and *f*2 = 48.12).

During the dissolution analyses of EFA 2, some residue was observed deposited at the bottom of the dissolution vessel, which may have been caused by a change in the crystal packing structure, for example hydrate formation that presented lower solubility and consequently leading to precipitation. 

**Figure 3:**
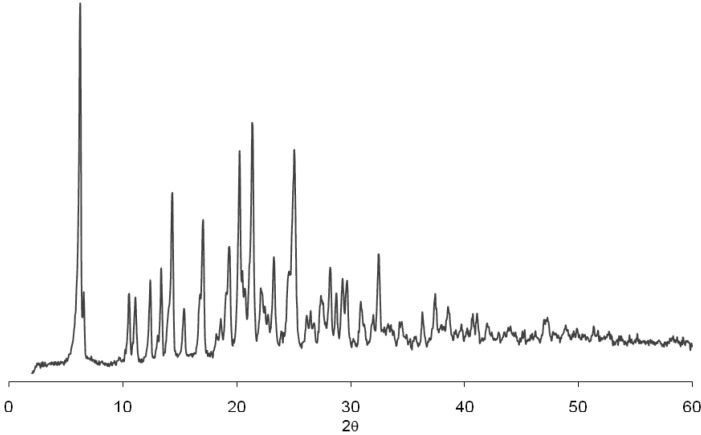
Diffraction patterns of dissolution residue of efavirenz batch 2 (EFA 2).

**Figure 4:**
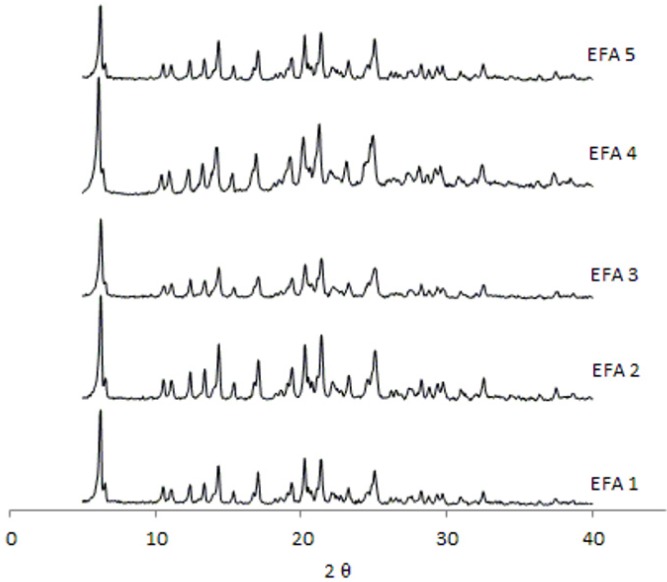
Diffraction patterns obtained from the efavirenz batches 1 to 5 (EFA 1 to 5).

To assess this possible transition, the residues of 3 vessels were separated by filtration and dried in vacuum desiccators and analyzed by XRD and NMR. Figure 3 shows the diffraction patterns of this residue and demonstrates no alteration in the physical conformation in comparison with the diffraction pattern of the pure drug (Figure 4). The analysis of ^1^H and ^13^C NMR (data not shown) of efavirenz batches EFA 1–5 clearly showed that the residues relate to efavirenz structure without impurities. So, this possibility can be excluded from the alternatives elected for this observation.

**Figure 5:**
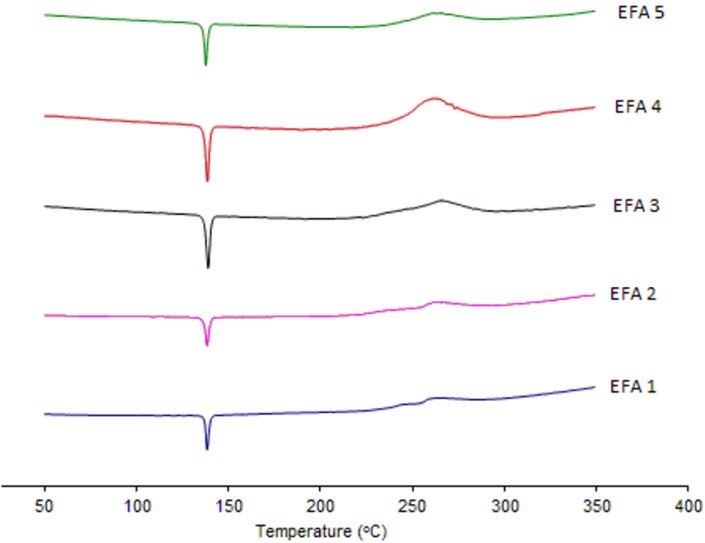
DSC curves of efavirenz batches 1 to 5 (EFA 1 to 5).

Analyses by XRD (Figure 4) and DSC (Figure 5) were also undertaken in order to evaluate the crystal structure of efavirenz batches 1 to 5. The DSC curves demonstrate the same thermal profile in all samples tested. The peaks related to crystal melting are at the same temperature and the enthalpies of those peaks are similar. The diffraction patterns (Figure 4) of all samples tested have, in the same way, very similar profiles. Therefore, the differences observed in dissolution of the API cannot be attributed to a difference in the crystalline structure of the drug.

The XRD analyses of all batches studied correspond to the structure of efavirenz isoform I [9], and there is no evidence of the presence of other polymorphs of the drug in those samples. No other transitions were observed, which indicates the absence of amorphous portions in the samples. The peaks corresponding to the structure of efavirenz isoform I are present, and no patterns indicating the presence of other crystalline structures in the samples were identified. When comparing these results with efavirenz synthesis patent [9], it can be seen that form I appears to be present in all batches. Therefore, differences in dissolution of the API cannot be attributed to a change in the crystalline structure of the drug (such as polymorphism, hydration or amorphization), but are instead due to the variation of size distribution and polydispersity index that should be controlled in the manufacturing process of the API [7,14].

A better correlation between the particle size of the API and dissolution can be obtained by measuring the surface area [17] using the BET equation. Table 3 shows a correlation between the surface area, the percentage dissolved and the size distribution of efavirenz batches 1–5. A significant difference between them can be seen, showing a correlation between particle size polydispersity index and surface area. It can be noted that the micronization process has a direct influence on the dissolution properties of the API. The dissolution profile of the efavirenz tablets produced (Table 1), demonstrates that micronization and the granulation process have a direct influence on solubilization and dissolution of the drug. 

**Table 3:** Values of surface area, percentage dissolved and size distribution of efavirenz batches 1–5.

Batch	Surface area Mean (m^2^/g) ± DPR	% dissolved in 60 min	*d*_(0.5)_ μm *	PI
EFA1	2.739 ± 4.7%	67.17 ± 3.5%	11.656	2.7513
EFA2	3.894 ± 0.02%	64.05 ± 2.4%	6.166	4.4301
EFA3	5.230 ± 0.1%	77.52 ± 3.3%	7.699	3.0375
EFA4	5.068 ± 1.3%	85.7 ± 3.9%	3.443	6.4139
EFA5	6.258 ± 1.0%	92.1 ± 2.1%	2.736	2.4412

* *d*_(0.5)_ μm: particle diameter corresponding to 50% of the cumulative distribution; PI refers to the polydispersity index.

It should be taken into consideration that the geometry of the particle is not controlled in the process of micronization, leading to particles of less spherical geometry and larger variability of measurement between samples. Again, it appears that the micronization process has a direct influence on the increase of the surface area and that the percentage of the API’s particles with a diameter less than 1.0 µm affects the results obtained. Mosharraf & Nyström [18], after evaluating parameters such as particle size and surface shape, observed that there is no direct correlation between micronization and increased dissolution. However, their research did not take into account the polydispersity index and particle surface area, which have been evaluated in this study. The data taken together show a direct relationship between the micronization technique used and dissolution of the drug studied. Vogt, Kunath & Dressman have demonstrated a significant increase in dissolution after the micronization of fenofibrate [14], contradicting Mosharraf & Nyström’s hypothesis [18]. The experiments were conducted with Alpine air blast equipment. The reduction in particle size by this process was substantial, almost 10-fold for the parameter *d*_(0.9)_. On the other hand, the values of *d*_(0.5)_ and particularly T_(0.1)_ did not change greatly. Kornblum & Hirschorn have demonstrated that micronization may create a tendency for large aggregates to form, leading to low dissolution [19]. These results indicate again the need for complete characterization in order to establish unequivocally the influence of particle size distribution on the technological results of the micronization process. The evaluation of only the crystalline structure of the micronized drug in the study of the solubilization of the API was a major constraint of these findings. The formulation of a batch of API obtained by an optimized micronization technique seems to be a good choice for this situation. Yet, the effect of the presence (or not) of other substances that may sterically prevent the agglomeration process due to greater lipophilicity should be considered. This can be observed in formulations containing a mixture of the API with other excipients [20].

Batch EFA 5 was chosen for the preparation of tablets in order to test the hypothesis that the excessive reduction of particle size of the API can lead to a partial agglomeration and collapse of the material, which may retard its dissolution [21]. Thus, the formulation described here (Table 1) was used for the preparation of tablets by wet granulation, with the direct application of water as the granulation liquid and the administering of this solvent in high-shear spray form. In the case of the tablets obtained by traditional granulation, drug dissolution after 30 min was 69.5% ± 3.4%, demonstrating the effect of the collapsing of the material and dissolution reduction due to the excess of added water. These tablets had an average content of 98.0% ± 0.2% and a hardness of 120 ± 2.3 N. However, with the tablets obtained by the high-shear mixer granulator process, the extent of dissolution after 30 min was 88.5% ± 1.6%, with an average content of 97.5% ± 0.3% and a hardness of 138 ± 1.4 N, leading to tablets suitable for marketing. These results indicate the need for strict control of the way that water is applied during granulation in order to obtain an adequate dissolution profile of the tablets produced, demonstrating that micronization leads to increased dissolution of the API, and that possible agglomeration could be prevented by optimizing the granulation process.

This hypothesis was supported by the formulation of batch EFA 2 using the high-shear spray granulation technique, which was considered more appropriate when the tablets were prepared using EFA 5. The value observed for the dissolution of the tablets EFA 2 was of 66.2% ± 1.5%, confirming the hypothesis of the influence of micronization on the success of the formulation of brick dust drugs such as efavirenz.

## 4. Conclusions

Based on these results, a direct relationship can be observed between the control of the micronization process of brick dust drugs and the particle size distribution and surface area, ensuring the possibility of converting these drugs into tablets or capsules with adequate bioavailability. The risk of partial agglomeration and collapsing of tablets formulated using APIs with very small particle size proved to be factors that may affect the formulation of efavirenz and other drugs with similar physical and chemical properties. This indicates the need for strict control of the processes used in the production of solid oral formulations. It also demonstrates that it is not only polymorphism that influences the solubility of a drug. Consequently, the control and proper characterization of the effect of particle size distribution on the dissolution of APIs proves to be a much bigger challenge for the manufacturing technology of solid oral dosage forms than has been believed up until now.
